# Exosome miR‐27a‐3p secreted from adipocytes targets ICOS to promote antitumor immunity in lung adenocarcinoma

**DOI:** 10.1111/1759-7714.13411

**Published:** 2020-03-25

**Authors:** Xuehan Fan, Jingya Wang, Tingting Qin, Yujia Zhang, Wenting Liu, Kaiting Jiang, Dingzhi Huang

**Affiliations:** ^1^ Department of Thoracic Oncology National Clinical Research Center for Cancer, Tianjin Key Laboratory of Cancer Prevention and Therapy, Tianjin’s Clinical Research Center for Cancer, Tianjin Medical University Cancer Institute and Hospital Tianjin China

**Keywords:** Exosome, ICOS, immunotherapy, lung adenocarcinoma, miR‐27a‐3p, obesity

## Abstract

**Background:**

The clinical benefit of immunotherapy has been limited to a small subset of patients with cancer. Several clinical trials with immune checkpoint inhibitors in multiple cancers have shown some improvement in obese patients. However, how obesity regulates the immune microenvironment remains unclear.

**Methods:**

Bioinformatic analysis was used to discover immune microenvironmental‐related genes associated with body mass index (BMI). The expression of ICOS in tumor tissues was detected using western blot, immunohistochemistry, quantitative real‐time polymerase chain reaction (RT‐qPCR) and flow cytometry. RT‐qPCR was used to measure the expression of miR‐27a‐3p. The interaction between miR‐27a‐3p and ICOS was confirmed by dual‐luciferase reporter assay. Functional testing of T cells based on proliferation and interferon (IFN)‐gamma secretion was performed using ELISA and flow cytometry.

**Results:**

ICOS, an immune microenvironment‐related gene, was significantly upregulated in obese patients with lung adenocarcinoma (LUAD). MiR‐27a‐3p showed a negative correlation with ICOS and suppressed the expression of ICOS. We determined that dipocyte‐derived exo‐miR‐27a‐3p could alter the tumor microenvironment by inhibiting ICOS^+^ T cell proliferation and IFN‐gamma secretion in vitro.

**Conclusions:**

Adipocyte‐derived exo‐miR‐27a‐3p can inhibit ICOS^+^ T cell proliferation and IFN‐gamma secretion. The upregulation of ICOS^+^ T cell functions caused by the downregulation of miR‐27a‐3p in adipose tissue derived exosomes is one of the potential mechanisms for the improved efficacy of immunotherapy in obese LUAD patients.

## Introduction

Lung cancer remains the predominant cause of cancer incidence and mortality worldwide. In addition, it is the leading cause of cancer death.^1^ Over the past decade, emerging therapeutic strategies, including targeted agents and immune checkpoint inhibitors, have altered the treatment paradigm of non‐small cell lung cancer (NSCLC). The use of immunotherapy has led to long‐term remission in a few patients^2^ and has improved survival in certain malignancies. However, the clinical benefit has been limited to a subset of patients because of primary resistance, acquired resistance, and immune‐related toxicity, etc.^3^ Thus, identifying patients who could benefit the most from such therapies is currently a major task in order to save more lives.

Obesity is highly prevalent worldwide and can cause a variety of comorbidities, including type 2 diabetes mellitus and cardiovascular diseases.^4^, ^5^ It is well known that obesity increases the risk of autoimmunity and cancer^6^ and is thought to promote tumor growth and T cell exhaustion.^7^, ^8^ Recently, several clinical trials with immune checkpoint inhibitors in a variety of cancers have observed a certain improvement in the clinical outcomes of obese versus nonobese patients.^9^, ^10^, ^11^ Obesity drives immune exhaustion by leptin,^12^ but the exact mechanism between obesity and the improved efficacy of immunotherapy is still unclear. This is an unmet need, and the clarification of this mechanism will help patients choose the optimal immunotherapy in clinical practice.

The most critical characteristic of obesity is the over‐expansion of adipose tissue.^13^ Previous studies have demonstrated that adipocyte‐derived exosomes lead to the development of insulin resistance by activating adipose‐resident macrophages and secreting proinflammatory cytokines,^14^ which are important components of the tumor immune microenvironment. Exosomes could promote inflammation of remote recipient cells by carrying proinflammatory factors.^15^, ^16^ These studies indicate that adipocyte‐derived exosomes may affect the immune microenvironment of remote organs.

Obese individuals have more adipocyte‐derived exosomes because of the larger total adipose tissue volume. Exosomes act as messengers in intercellular communication because they contain multiple proteins, lipids, and miRNAs.^17^ MiRNAs are involved in a variety of biological processes, such as cell proliferation, differentiation, and immune responses.^18^ The functions of exosomal miRNAs originating from adipocytes have not been studied in the immune microenvironment in lung adenocarcinoma (LUAD). Therefore, we hypothesize that adipocytes shed exosomes containing miRNAs that activate the inflammatory microenvironment of remote organs to modulate the efficacy of immunotherapy.

Herein, we first demonstrate that inducible costimulatory molecule (ICOS) was upregulated in the tumor tissues of obese patients with LUAD. ICOS is an essential costimulatory molecule for T cell function. By comparing the expression patterns of miR‐27a‐3p and ICOS between LUAD patients with higher and lower body mass index (BMI), we found that miR‐27a‐3p expression was negatively correlated with ICOS expression in tumor tissues. The luciferase reporter assay showed that ICOS could be directly regulated by miR‐27a‐3p. We also determined that adipose‐derived exosomes from obese individuals obviously reduced the level of miR‐27a‐3p and promoted ICOS^+^ T cell proliferation and IFN‐gamma secretion. Thus, this finding offers new insight into why overweight patients derive a greater clinical benefit from immunotherapy than nonobese patients.

## Methods

### Gene expression datasets

This study made use of public domain level 3 data of gene expression profiles of LUAD patients downloaded from The Cancer Genome Atlas (TCGA) dataset (April 2019). Immune and stromal scores were calculated by ESTIMATE,^19^ an algorithm providing stromal scores and immune scores in tumor tissues. To analyze the relationship between obesity and gene expression profiling, we also collected the microarray data under accession number GSE103512 from the Gene Expression Omnibus (GEO) database, which was based on GPL13158 platforms (Affymetrix HT HG‐U133+ PM Array Plate). To analyze the relationship between obesity and visceral adipocyte‐derived exosomal miRNAs, GSE50574 microarray data were collected from the GEO database, which was based on GPL16384 platforms [miRNA‐3] (Affymetrix Multispecies miRNA‐3 Array).

### Differential analysis of expressed genes

The Bioconductor package, “limma”, was used to identify differentially expressed genes (DEGs). According to the ESTIMATE results, all samples were divided into high‐ and low‐immune score groups and high‐ and low‐ stromal score groups to select the common genes. FDR < 0.05, logFC ≥ 1 and *P*‐value <0.05 were set as the cutoff criteria. Moreover, all samples from GSE103512 were divided into an overweight group (BMI ≥ 25 kg/m^2^) and normal‐weight group (BMI < 25 kg/m^2^) to identify DEGs. Heatmaps were generated using the “pheatmap” package in R software.

### TIMER analysis

Gene expression profiles from the TCGA database were used to systematically analyze the tumor‐infiltrating immune cells using the Tumor IMmune Estimation Resource (TIMER) algorithm.^20^, ^21^

### Cell sorting and culture

The 3T3‐L1 murine preadipocyte cell line was purchased from the cell bank of the Chinese Academy of Sciences (Shanghai, China) and cells were maintained in DMEM/F12(Gibco) supplemented with 10% fetal bovine serum(FBS, Gibco) at 37°C with 5% CO_2_. To induce 3T3L1 cells into mature adipocytes, indomethacin (0.2 mM; Sigma), dexamethasone (1 μM; Sigma), isobutylmethylxanthine (0.5 mM; Sigma), insulin (10 μg/mL), and rosiglitazone (2.5 μM; Sigma) were added to DMEM/F12(Gibco).^22^ Lymphocytes were isolated from the spleen of a C57/BL6 mouse using the Myltenyi negative selection kit. C57/BL6 female mice, purchased from SPF (Beijing) Biotechnology Company, aged 6–8 weeks were raised under specific pathogen‐free conditions. Lymphocytes were cultured in RPMI 1640(Gibco) supplemented with 10% fetal bovine serum at 37°C with 5% CO_2._ Lymphocytes were activated with anti‐mouse CD3ε(14–0031‐82, eBioscience) antibody and anti‐CD28(16‐0281‐81, eBioscience) according to the manufacturer's protocols.

### Cell transfection

The miR‐27a‐3p mimics, inhibitor and the negative control synthesized by Gene Pharma (Shanghai, China) were transfected into cells using Lipofectamine 3000 Reagent (Invitrogen, California, USA), according to the manufacturer's specifications. The culture medium was changed six hours after transfection.

### Isolation of exosomes from plasma and medium

The method for the ultracentrifugation of exosomes from plasma and medium was based on previous reports.^23^ The medium was initially passed through a 0.2 μm aperture pore filter, and then ultracentrifuged at 150 000 × g overnight at 4°C. Finally, the exosome pellet was resuspended in PBS and centrifuged again at 150 000 × g at 4°C for two hours. Exosomes were resuspended in PBS for protein analyses or in 500 μL of TRIzol (Invitrogen, California, USA) for RNA analyses.

### Transmission electron microscopy assay

The exosomes were fixed with 4% paraformaldehyde and 4% glutaraldehyde in 0.1 M phosphate buffer (pH 7.2) at 4°C. Exosome samples were dropped onto EM grids and incubated for 20 minutes. The grid was blocked with glycine/PBS and 5% BSA/PBS for 10 minutes. The cells were washed three times in PBS. Samples were embedded on ice in 1% methylcellulose and 4% uranyl acetate. Finally, the grids were examined with a transmission electron microscope at 60–80 kV.

### RNA isolation and real‐time PCR

Total RNA of cells and exosomes was isolated using TRIzol Reagent (Invitrogen, California, USA) according to the manufacturer's protocol. Quantitative reverse transcription PCR (RT–qPCR) was performed with DNase‐treated RNA using the Mir‐X miRNA RT‐qPCR TB Green Kit (638314 Takara Bio USA) on an Applied Biosystems AB7500 Real‐Time PCR system. The delta‐delta Ct method was used to measure the relative levels of miRNA between two samples by normalizing them to U6 levels. The primers were as follows:

miR‐27a‐3p: Forward primer:5'TGCGGTTCACAGTGGCTAAG3'

Reverse primer:5'CTCAACTGGTGTCGTGGA3'

U6: Forward primer:5'CTCGCTTCGGCAGCACA3'

Reverse primer:5'AACGCTTCACGAATTTGCGT3'

ICOS: Forward primer:5'TTTGAACACTGAACGCGAGG3'

Reverse primer:5'CAGAACCATTGATTTCTCCTGTT3'.

### miRNA target prediction

The miRNAs that regulate the DEGs were predicted using the MicroRNA Data Integration Portal.^24^

### Luciferase assay

The ICOS 3'‐UTR was cloned into a pGL6‐miR luciferase gene expression vector (Beyotime, Wuhan, China). HEK‐293T cells were seeded into a 24‐well plate and cotransfected with miR‐27a‐3p mimics or miR‐27a‐3p mimics‐NC and pGL6‐miR ICOS 3'UTR WT/Mut using Lipofectamine 3000 (Invitrogen, California, USA). Luciferase activities were assessed using the Dual‐Luciferase Reporter Assay Kit (Beyotime Biotechnology, Shanghai, China) according to the manufacturer's protocol.

### Western blot

A total of 16 human LUAD tissue samples were obtained from Tianjin Medical University Cancer Institute and Hospital and informed consent was provided by all patients. Total protein was prepared in RIPA buffer (Solarbio, Beijing, China). Each sample was separated by a 10% SDS‐PAGE gel and transferred to a PVDF membrane. After blocking for two hours with 5% BSA, membranes were incubated overnight at 4°C with the primary antibodies, anti‐CD63 (Santa Cruz), anti‐tsg101 (Santa Cruz), anti‐ICOS (Abcam), and anti‐β‐actin (ZSGB‐BIO, China). The blots were incubated with secondary antibodies (ZSGB‐BIO, China) (1:2000) for one hour at room temperature, followed by detection with ECL reagents (Pierce). Protein levels were quantified using ImageJ software.

### Oil red O staining

After 3T3‐L1 preadipocytes were induced, intracellular lipids accumulated in the cells. The cells were washed twice with PBS, fixed with 4% formaldehyde at room temperature, and washed three times with PBS. The cells were treated with filtered Oil Red O solution for one hour at room temperature and then washed twice with PBS. The resulting red‐stained lipid droplets were observed under a microscope.

### Immunohistochemistry

We obtained paraffin‐embedded LUAD tissues from Tianjin Medical University Cancer Institute and Hospital collected between June 2016 and December 2018. Informed consent was provided by all patients. The tumors were fixed in 4% paraformaldehyde and embedded in paraffin. Sections were dewaxed and rehydrated, and peroxidase activity was blocked with 3% hydrogen peroxide solution. An anti‐ICOS antibody (CST89601,1:200) was added to the samples after incubation in a blocking solution. The staining score was calculated by multiplying the proportion of positive cells (0, ≤5%; 1, 6 ≤ 15%; 2, 16 ≤ 30%; 3, 31 ≤ 50%; 4, >50%) by the intensity of staining (0, negative; 1, weak; 2, medium; 3, strong). The protein expression was classified into four categories based on the final staining scores with “−” for a score of 0–3, “+” for a score of 4–6, “++” for a score of 7–9, and “+++” for a score of 10–12. High expression was defined as six points or more.

### ELISA

A total of 5 × 10^5^ splenocytes from C57/BL6 mice were cocultured with adipocyte‐derived exosomes for 72 hours in U‐bottom 96‐well plates. The supernatant was collected to perform ELISA experiments. ELISA quantification of IFN‐gamma in the culture medium was performed using a mouse IFN‐gamma ELISA kit (4A Biotech, Beijing, China) following the manufacturer's instructions.

### Flow cytometry

Blood samples were obtained from patients with LUAD before any treatments. We then used 100 μL of whole blood with a lysis protocol for staining. Whole blood was stained with the following fluorochrome‐conjugated monoclonal Abs: APC‐CD3(561810), FITC‐CD8(560960), PerCP‐Cy5.5‐CD4(552838), PE‐ICOS(557802) (all from BD Pharmingen, San Diego, CA) and relevant isotype controls for 30 minutes away from light. Whole blood was hemolyzed with lysis buffer followed by washing three times with PBS and then assayed on a FACSCanto (BD Biosciences). Flow cytometric analysis was performed with FlowJo software.

### Statistical analysis

Data were analyzed with GraphPad Prism 7. Experiments were repeated at least three times. Statistical analysis was performed using Student's *t*‐test or the Wilcoxon‐Mann‐Whitney test. One‐way analysis of variance was used to compare the immune and stromal scores in different groups. Differential analysis of expressed genes was performed using R version 3.5.3. *P* < 0.05 was considered statistically significant.

## Results

### ICOS is an immune microenvironmental‐related gene associated with BMI

**Figure 1:**
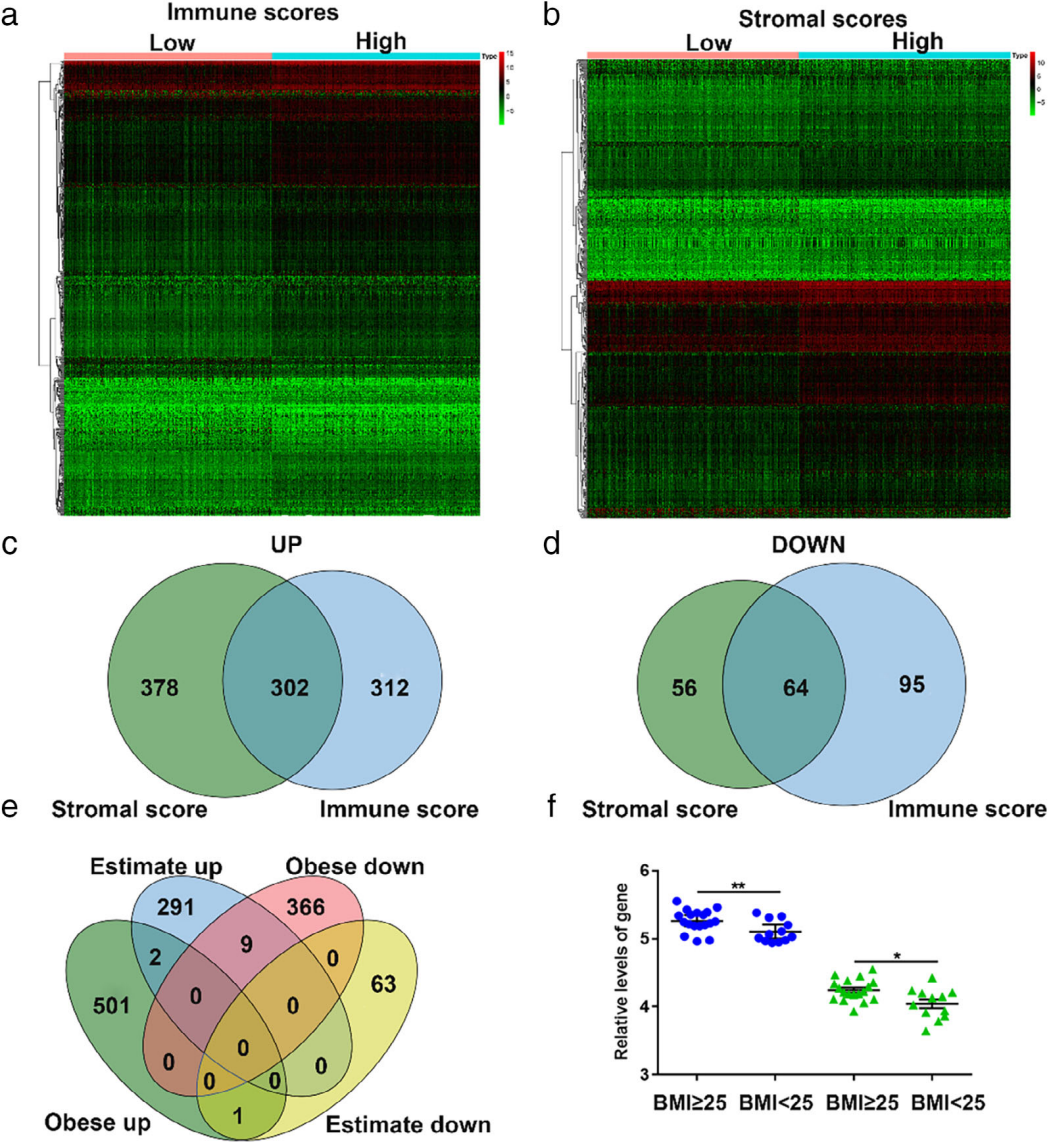
Comparison of gene expression profile with immune scores and stromal scores in LUAD. (**a**) Heatmap of the differentially expressed genes (DEGs) of immune scores of high scores versus low scores. *P* < 0.05, fold change >2). (**b**) Heatmap of the DEGs of stromal scores of high scores versus low scores. *P* < 0.05, fold change >2). (**c**, **d**, **e**) Venn diagrams showing the number of commonly upregulated (**c**) or downregulated (**d**) DEGs. (**f**) Relative levels of DEGs (**P < 0.01, *P < 0.05) (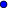) RSPO1, (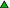) ICOS.

To reveal the correlation between gene expression profiles and immune/stromal scores, we performed the ESTIMATE algorithm on the expression profiles of all 533 LUAD patients in the TCGA database. The differentially expressed profiles of the high versus low immune/stromal score groups are shown in the heatmaps (Fig 1a,b). There were 614 upregulated genes and 159 downregulated genes in the high immune score group and 680 upregulated genes and 120 downregulated genes in the high stromal score group (Fig 1c,d). Moreover, the Venn diagram (Fig 1c,d) showed that there were 302 commonly upregulated genes and 64 downregulated genes between the high score groups.

We used the same method to explore the relationship between gene expression profiles and obesity. Patients from GSE103512 were classified into the obese group and normal group by their BMI. There were 504 upregulated genes and 64 downregulated genes in the obese group (Fig 1e). Finally, we obtained two BMI associated immune microenvironmental‐related genes, ICOS and Rspo1, as described in the Venn diagram (Fig 1e). The results showed that ICOS and Rspo1 levels were significantly higher in the overweight group (Fig 1f). Circulating Rspo1 levels were reported to be increased to a greater extent in the obese group than in the lean group,^25^ while in another study anti‐Rspo1 antibody together with an anti‐PD‐1 antibody had an enhanced antitumor effect.^26^ The result suggested that Rspo1 was not a direct synergistic gene for immunotherapy in obese patients. Therefore, we focused on the relationship between ICOS and obesity.

### Comparison of expression levels of ICOS in relation to obesity and baseline characteristics of participants

**Figure 2:**
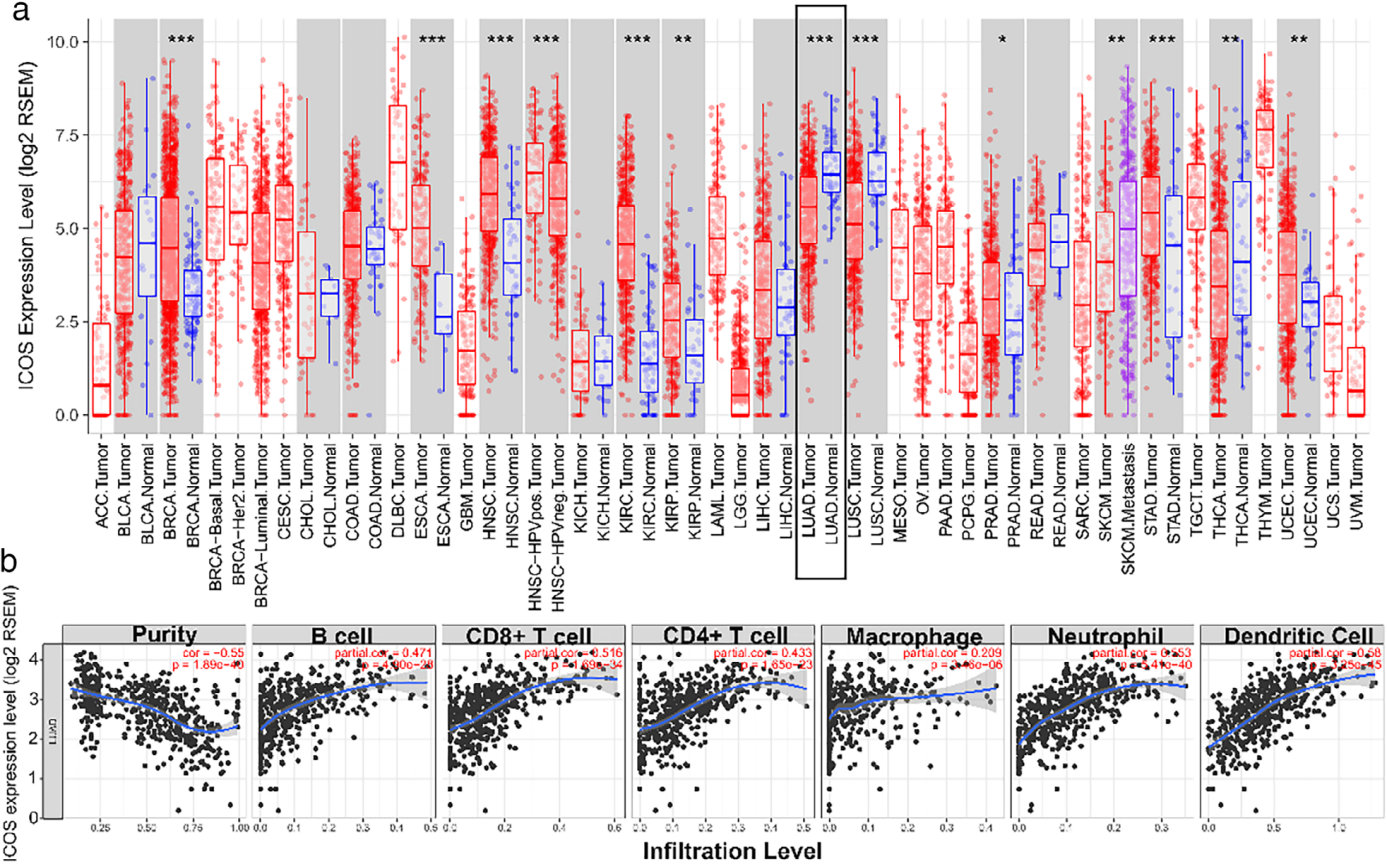
(**a**) The level of ICOS expression in tumor and normal tissue from the TCGA database in TIMER (*P < 0.05, **P < 0.01, ***P < 0.001). (**b**) Correlation analysis of ICOS expression and infiltration levels of immune cells in LUAD using the TIMER database.

Gene expression analyses of TCGA RNA‐seq data using the TIMER database showed that the ICOS mRNA levels were lower in LUAD compared with the corresponding normal tissue (Fig 2a). We explored the relationship between ICOS expression and the infiltrating immune cells in LUAD using the TIMER database. The results showed that ICOS expression was significantly correlated with tumor purity in LUAD. Moreover. ICOS expression was significantly correlated with the infiltration levels of CD8^+^ T cells, CD4^+^ T cells, B cells and dendritic cells (Fig 2b). This result indicated that ICOS expression was closely related to the immune system.

**Figure 3:**
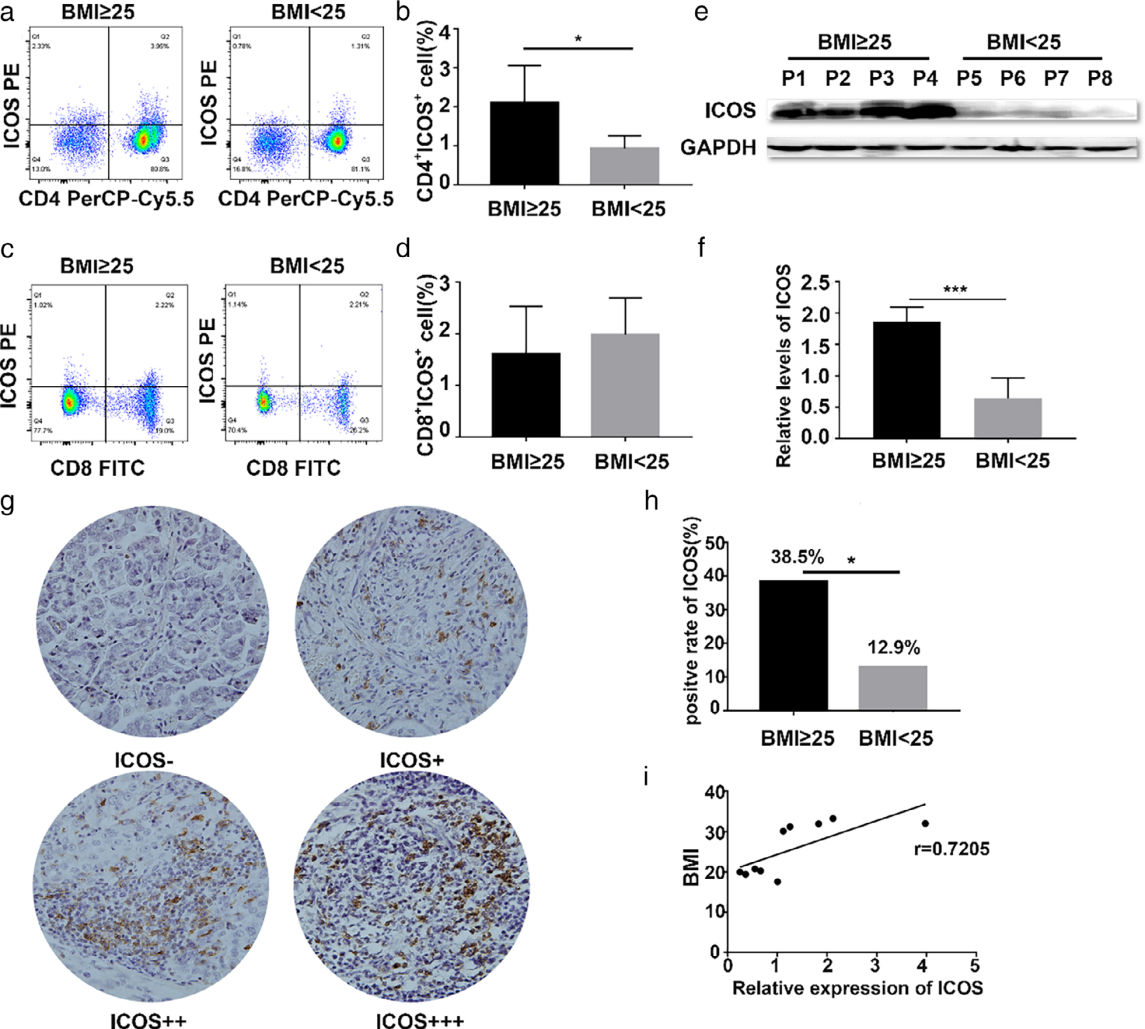
The correlation between ICOS and BMI in LUAD. (**a**, **b**) The ratio of CD4^+^ICOS^+^ T cells in peripheral blood from patients by flow cytometry (*P < 0.05). (**c**, **d**) the ratio of CD8^+^ICOS^+^ T cells in peripheral blood from patients by flow cytometry. (**e**, **f**) Western blot analysis of ICOS in above the median group (*n* = 11) and below the median group (*n* = 13) (***P < 0.001). (**g**,**h**) Immunohistochemical analysis of the paraffin‐embedded tumor tissues using an ICOS antibody in different BMI groups (*n* = 26 and *n* = 31, respectively) and the corresponding quantification (*P < 0.05) (original magnification 100×). (**i**) RT‐qPCR analysis the correlation between ICOS expression and BMI in tumor tissues(n = 10).

Subsequently, we measured ICOS expression in peripheral blood and tumor tissues of both groups. Peripheral blood was collected from LUAD patients before any treatment. As shown in Fig 3a–d, we observed that obese LUAD patients contained two‐fold more CD4^+^ICOS^+^ T cells than normal‐weight patients, but there was no difference in CD8^+^ICOS^+^ T cells. ICOS expression at the protein level in tumor tissue was also quantitatively analyzed by western blot and the staining intensity results were quantified. As expected, the expression level of ICOS was also clearly higher in the obesity group than in the normal group. (Fig 3e,f). We then detected the expression of ICOS in tumor tissues by immunohistochemistry (IHC) (Fig 3g). ICOS staining was performed in 58 patients with primary LUAD and was found to be mainly located in the membrane region in tumor tissues. Representative staining of ICOS in LUAD is shown in Fig 3g. High level of ICOS staining was significantly correlated with BMI (Fig 3h) but not with other parameters, such as gender, age, stage, and histological subtype. Specific data are shown in Table 1. Finally, the mRNA expression levels of ICOS were detected by RT‐qPCR. As shown in Fig 3i, the mRNA expression of ICOS showed a positive correlation with BMI.

**Table 1:** Stratified analysis of ICOS expression in lung adenocarcinoma

Factors	n	Cytomembrane low group	Cytomembrane high group	*P*‐value
Gender				0.249
Male	25	17 (68%)	8 (32%)	
Female	32	26 (81.3%)	6 (18.8%)	
BMI				0.026
≥25	26	16 (61.5%)	10(38.5%)	
<25	31	27 (87.1%)	4(12.9%)	
Age, years				0.792
<70	50	38 (76%)	12 (24%)	
≥70	7	5 (71.4%)	2 (28.6%)	
T				0.826
T1	34	26 (76.5%)	8 (23.5%)	
T2–4	23	17 (73.9%)	6 (26.1%)	
Lymph node metastasis				0.408
Absent	42	30 (71.4%)	12 (28.6%)	
Present	15	13 (86.7%)	2 (13.3%)	
Distant metastasis				0.057
Absent	55	43 (78.2%)	12 (21.8%)	
Present	2	0 (0.0%)	2 (100%)	
Histological subtype				0.464
Micropapillary/solid	41	32 (78.0%)	9 (22.0%)	
Others	16	11 (68.8%)	5 (31.3%)	
Stage				0.948
I	33	25 (75.8%)	18 (75.0%)	
II–IV	24	8 (24.2%)	6 (25.0%)	

### MiR‐27a‐3p downregulated in adipocyte‐derived exosomes from obese people and could directly interact with ICOS

Obesity is a systemic inflammatory state^27^ associated with chronic inflammatory status impacting nearly every organ system in the body.^28^ Previous studies have shown that adipocyte‐derived exosomes from obese individuals contain miRNAs that activate remote organ inflammation.^29^ Obese individuals have a much larger overall adipose volume than lean individuals and thus the relative amount of shed adipocyte‐derived exosomes would also likely be greater.

**Figure 4:**
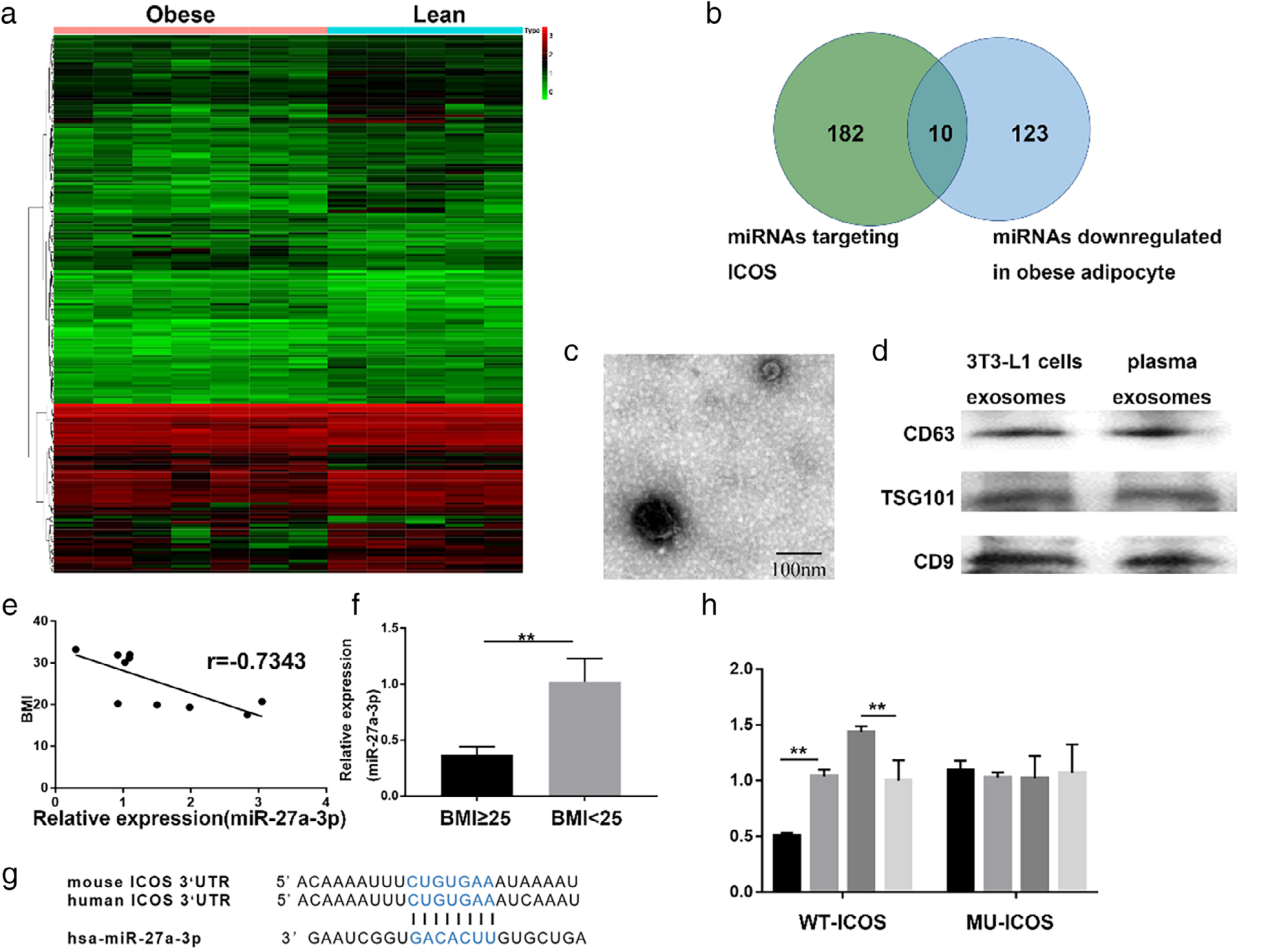
Validation of ICOS as a direct target of miR‐27a‐3p. (**a**) Heatmap of differentially expressed exosomal miRNAs in the GSE50574 dataset, which included visceral adipose tissue samples obtained from patients with obesity and lean patients. (**b**) The Venn diagram revealed the intersection of miRNAs targeting ICOS and miRNAs downregulated in obese visceral adipose tissues. (**c**) Transmission electron microscopy image of exosomes (scale bar, 100 nm). (**d**) Plasma exosomes were isolated from LUAD patients. Western blotting of three representative exosome specific markers: CD63, CD9, and tsg101. (**e**) RT‐qPCR analysis the correlation between miR‐27a‐3p expression and BMI in tumor site (*n* = 10). (**f**) PCR analysis of miR‐27a‐3p in exosomes of peripheral blood (**P < 0.01). (**g**) Putative binding sites for miR‐27a‐3p and ICOS. (**h**) Luciferase reporters containing either wild‐type or mutant ICOS, miR‐27a‐3p mimics and the normal control were cotransfected into HEK293 T cells. The relative luciferase levels were detected after transfection (**P < 0.01) (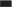) mimics, (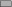) mimics NC, (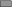) inhibitors, (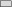) inhibitors NC.

To further explore the cause of the high expression of ICOS in obese people, we analyzed the differentially expressed exosomal miRNAs (DE‐miRNAs) in the GSE50574 dataset. According to the threshold criterion of *P* < 0.05, 189 DE‐miRNAs were identified, of which 133 were downregulated and 56 were upregulated (Fig 4a).

MiRNA target prediction analyses were performed with algorithms from the MicroRNA Data Integration Portal, which contains multiple independent microRNA prediction databases. We found a series of miRNAs targeting ICOS. A Venn diagram analysis revealed 10 shared miRNAs (Fig 4b). MiR‐27a‐3p was found to be the only miRNA with very high predicted confidence. We subsequently measured miR‐27a‐3p expression in tumor tissues. As demonstrated in Fig 4e, miR‐27a‐3p showed a significantly negative correlation with BMI (r = −0.7343). To further investigate the function of circulating exosomes carrying miRNAs in LUAD patients, we purified exosomes from plasma collected from patients before any treatments. Exosomes were characterized by TEM (Fig 4c), and were found to express conventional exosome markers: CD63, CD9, and tsg101 (Fig 4d). RT‐qPCR results showed that the expression level of miR‐27a‐3p carried by plasma exosomes increased nearly three‐fold in normal‐weight patients compared with obese patients, indicating that plasma exosomal miR‐27a‐3p is downregulated in the obese group and is closely related to the expression of ICOS in LUAD.

As shown in Fig 4g, miR‐27a‐3p could bind a highly conserved, complementary site in the 3'UTR of ICOS mRNA. We performed a luciferase reporter assay with a vector containing the wild‐type or mutated 3'UTR of ICOS (WT‐ICOS or MU‐ICOS) to determine whether ICOS was a direct target of miR‐27a‐3p. WT‐ICOS 3'UTR luciferase activity was inhibited in the miR‐27a‐3p mimics group as compared with the others, and MU‐ICOS 3'UTR luciferase activity was not changed(Fig 4h). These results indicate that miR‐27a‐3p directly suppresses ICOS.

### Upregulation of adipocyte exo‐miR‐27a‐3p inhibits ICOS^+^ T cell proliferation and IFN‐gamma secretion

**Figure 5:**
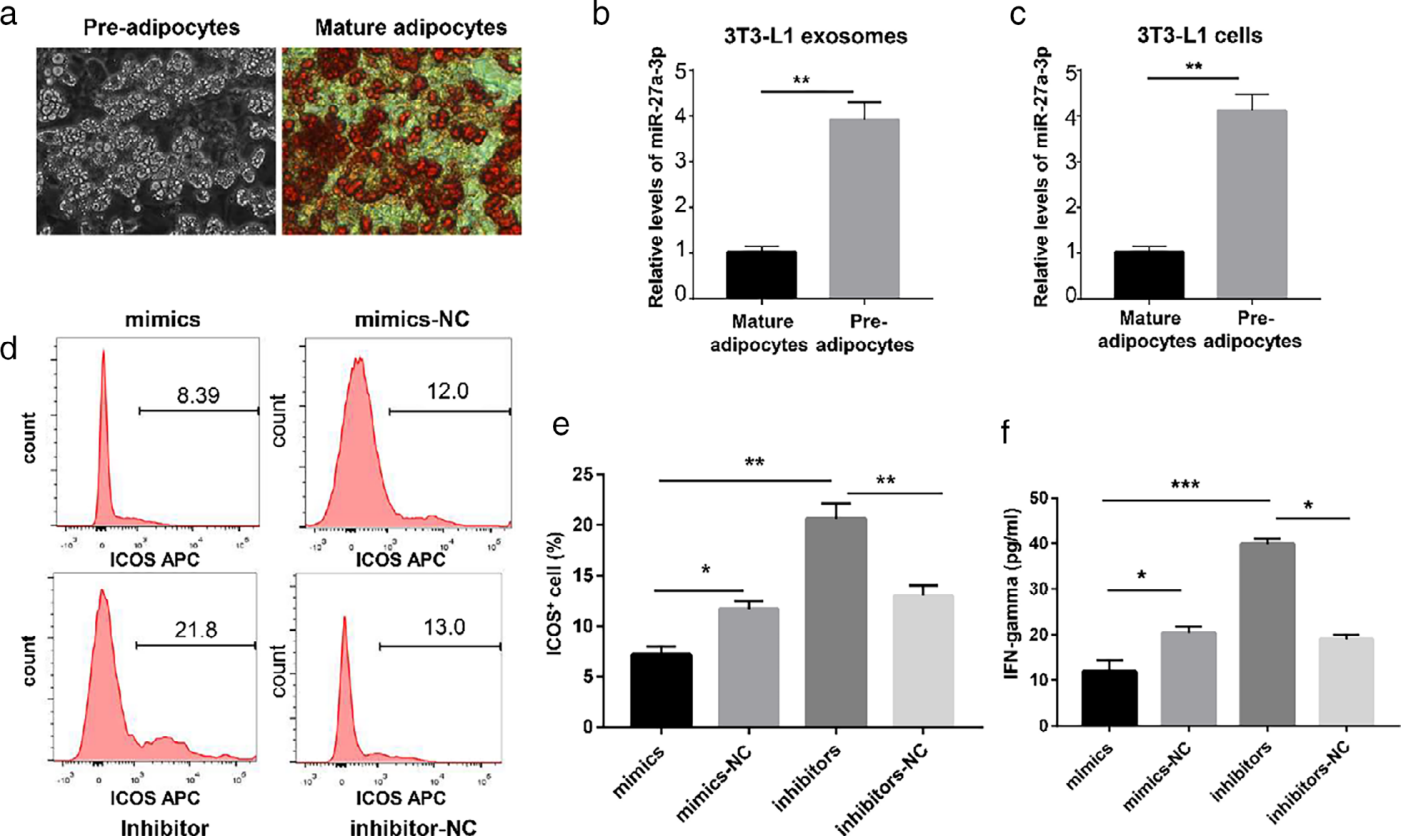
The expression of miR‐27a‐3p and ICOS is regulated by adipocyte exosomes. (**a**) Oil red O staining of mature adipocytes and pre‐adipocytes. (**b**, **c**) The difference in miR‐27a‐3p expression in mature and preadipocytes. (**P < 0.01) (**d**, **e**) Determination of ICOS^+^ T cell proliferation by flow cytometry. (*P < 0.05, **P < 0.01) (**f**) Determination of IFN‐gamma secretion by ELISA. (*P < 0.05, **P < 0.01, ***P < 0.001).

It is known that adipose tissue has different biological functions in different stages of differentiation. The oil red staining assay showed that intracellular lipids accumulated in mature adipocytes but not in preadipocytes (Fig 5a). To analyze the expression of miR‐27a‐3p in different stages of adipose tissue differentiation, we isolated exosomes from the supernatant of 3T3‐L1 cells. The exosomes secreted by 3T3‐L1 cells had high expression of CD63, CD9, and tsg101(Fig 4d). Compared with those in preadipocytes, exo‐miR‐27a‐3p expression levels were significantly decreased in mature adipocytes, and the same results were observed at the cellular level (Fig 5b,c).

Costimulatory receptors such as ICOS function by inducing higher T cell activation after TCR stimulation. To determine whether exo‐miR‐27a‐3p was able to induce costimulation, we used two independent functional T cell assays based on proliferation and IFN‐gamma secretion.

To explore the distinctive functions of adipocyte‐derived exosomal miR‐27a‐3p in T cell function, 3T3‐L1 cells were transfected with siRNAs targeting miR‐27a‐3p, and then the supernatants containing exosomes were separated to coculture with lymphocytes seeded in six‐well plates that were isolated from spleen and activated with anti‐CD3 agonistic antibody and anti‐CD28 antibody. The results revealed that T lymphocytes incubated with adipose exo‐miR‐27a‐3p inhibitors exhibited a higher ICOS^+^T cell rate than the controls, as measured by flow cytometry (Fig 5d,e).

Another indicator of T cell function is the secretion of IFN‐gamma. To that end, we stimulated purified T lymphocytes with a suboptimal amount of anti‐CD3 antibody and anti‐CD28 antibody. In addition, we added adipose exo‐miR‐27a‐3p as a co‐stimulus. T lymphocytes cocultured with inhibitors of miR‐27a‐3p, in all groups, produced the most amount of IFN‐gamma, which was especially highlighted by a more than two‐fold increase compared to the control groups (Fig 5f).

## Discussion

Obesity is an independent risk factor for certain malignancies and is characterized by a metainflammatory state and disordered immune responses.^7^ Recently several studies have reported that obesity is associated with higher efficacy of immune checkpoint inhibitors in both tumor‐bearing mice^9^ and clinical cancer patients.^9^, ^10^, ^11^ The mechanism by which obesity, which promotes tumor progression, can make immunotherapy in tumor patients more effective is unclear. Clarifying this mechanism will help match patients with the most effective immunotherapy.

To explore the underlying mechanisms, we identified a gene, ICOS, that is related to both BMI and the immune microenvironment. ICOS, known as a marker of T cell activation,^30^ is an essential costimulatory molecule for T cell enhancement.^31^, ^32^ Yi *et al*. have reported that T cells from peripheral blood and tumor tissues of patients treated with anti‐CTLA‐4 for NSCLC had an increased expression of ICOS.^33^ ICOS upregulation on T cells is correlated with increased clinical responses induced by CTLA‐4 inhibition.^34^, ^35^ Therefore, ICOS agonist mAbs could enhance the effect of inhibitory checkpoint inhibitors. Three ICOS‐specific agonistic mAbs are currently used alone or in combination with anti‐PD‐L1 to treat advanced solid tumors including NSCLC.^36^, ^37^ However, none of the described approaches have explored the correlation between ICOS and obesity.

Herein, we first determined that ICOS is significantly upregulated in obese LUAD patients. Through multiparametric flow cytometry, we found increased ICOS expression in CD4^+^ T cells in obese patients but surprisingly no difference in CD8^+^ T cells. Despite the small sample size, this finding indicates that CD4^+^ICOS^+^ T cells play an important role in affecting the efficacy of immunotherapy in obesity. Therefore, our work suggests that high‐load adipose cells in obese patients may affect CD4^+^ T helper cell function through the ICOS pathway, playing a role in immunotherapy. Pretherapeutic infiltration of CD8^+^ T cells in tumor tissues has been demonstrated to be correlated with the response to immunotherapy in tumor patients.^38^ A previous study revealed that in NSCLC patients receiving nivolumab, high numbers of peripheral CD8^+^ T cells lacking costimulatory receptors (CD28, ICOS, OX40) at baseline are associated with the response.^39^ Our findings suggest that obesity may not affect the function of peripheral CD8^+^ T cells through the ICOS pathway.

Adipose‐derived exosomes containing various circulating miRNAs regulate gene expression in distant tissues.^17^ The role of adipose‐derived exosomal miRNAs in the immune microenvironment in LUAD has not yet been reported, and here, we induced 3T3‐L1 cells into mature adipocytes and found that miR‐27a‐3p is downregulated in exosomes secreted by mature adipocytes, which is consistent with previous bioinformatic results. Moreover, we observed that not all exosomes in peripheral blood were secreted by adipocytes. Thus, we also performed experiments investigating the effects of adipose‐derived exo‐miR‐27a‐3p on T cells in vitro.

The upregulation of ICOS mediated by adipose‐derived exo‐miR‐27a‐3p plays a key role in promoting the secretion of IFN‐gamma. IFN‐gamma is an important activator of innate and adaptive immunity, with immunostimulatory and immunomodulatory effects that enhance the antitumor immune response.^40^ In addition, Overacre *et al*. indicated that IFN‐gamma‐induced Treg fragility is required for effective PD1‐targeted immunotherapy.^41^ Our work suggests that obesity plays a role in immunotherapy through immune‐assisted pathways.

However, the sample size in our study was small and the relationship between ICOS, obesity, and improved efficacy was not directly explored in patients receiving immunotherapy. In the meantime, it appears that adipose tissue metabolism is related to the immune microenvironment of tumor patients, and therefore a well‐designed experiment is needed to explore whether it is possible to enhance the efficacy of immunotherapy by regulating fat metabolism.

In conclusion, our work demonstrates that adipose tissue can promote ICOS expression by downregulating adipose‐derived exo‐miR‐27a‐3p and thereby influence IFN‐gamma secretion which is required for effective immunotherapy. These findings provide new insight into the association between obesity and improved immunotherapy efficacy. MiR‐27a‐3p and ICOS could be potential targets for improving the efficacy of immunotherapy.

## Disclosure

The authors declare that they have no conflicts of interest in this work.
